# The Role of Tumor Microenvironment and Targeted Therapy in Chronic Lymphocytic Leukemia

**DOI:** 10.3390/cimb47080604

**Published:** 2025-08-01

**Authors:** Khalil Saleh, Ahmadreza Arbab, Nadine Khalife, Rita Khoury, Rebecca Ibrahim, Mohamad Ali Hachem, Cynthia Khalil, Cendrella Bou Orm, Joud Sawan, Geoffroy Lafarge, Nohad Masri, Zamzam Tikriti, Claude Chahine, Axel Le Cesne

**Affiliations:** 1 International Department, Gustave Roussy Cancer Campus, 94800 Villejuif, France; rita.khoury011@gmail.com (R.K.); rebecca.ibrahim@gustaveroussy.fr (R.I.); mohamadali.hachem@gustaveroussy.fr (M.A.H.); cynthia.khalil@gustaveroussy.fr (C.K.); cendrella.bou-orm@gustaveroussy.fr (C.B.O.); joud.sawan@gustaveroussy.fr (J.S.); nohad.masri@gustaveroussy.fr (N.M.); zamzam.tikriti@gustaveroussy.fr (Z.T.); claude.chahine@gustaveroussy.fr (C.C.); axel.lecesne@gustaveroussy.fr (A.L.C.); 2 Department of Biopathology, Gustave Roussy Cancer Campus, 94800 Villejuif, France; ahmadreza.arbab@gustaveroussy.fr (A.A.); geoffroy.lafarge@aphp.fr (G.L.); 3 Department of Head and Neck Oncology, Gustave Roussy Cancer Campus, 94800 Villejuif, France; nadine.khalife-saleh@gustaveroussy.fr

**Keywords:** tumor microenvironment, BTK, BCL-2, CAR T-cell, bispecific, chronic lymphocytic leukemia

## Abstract

Chronic lymphocytic leukemia (CLL) is the most common leukemia in adults. It is characterized by the clonal proliferation of mature B cells. The tumor microenvironment (TME) seems to play a crucial role in the survival and proliferation of tumor cells. Multiple new classes of drugs had been approved for the management of patients with CLL, reshaping the treatment paradigm. The most important classes are Bruton’s tyrosine kinase (BTK) inhibitors and BCL-2 inhibitors. Both of them are approved as a first-line treatment in patients with CLL requiring treatment. The role of BTK and BCL-2 in the signaling pathways of the TME is very important. The aim of this review is to summarize the major components of the TME and the available data regarding targeted therapies in CLL.

## 1. Introduction

Chronic lymphocytic leukemia (CLL) is an indolent hematological neoplasm characterized by the clonal expansion and proliferation of mature B cells. It is the most frequent leukemia worldwide, with an estimated incidence of 100,000 new cases of CLL in 2019 and nearly 44,000 deaths [1,2]. This malignancy is mainly diagnosed in elderly patients, with a median age at diagnosis of more than 70 years. Of note, the incidence of CLL is five to ten times higher in the USA and Europe compared with Asian countries [3,4,5]. The treatment landscape of CLL has dramatically changed over the last decade with the advent of two classes of therapy: Bruton’s tyrosine kinase (BTK) inhibitor and B-cell leukemia/lymphoma-2 (BCL-2) inhibitors. Ibrutinib, a first-generation BTK inhibitor, is the first targeted therapy approved for the management of patients with relapsed and/or refractory (R/R) CLL in February 2014, with an overall response rate (ORR) of 71% [6]. Ibrutinib was then approved as first-line treatment with impressive results in comparison with the standard-of-care, with a median progression-free survival (PFS) not reached at 7 years of follow-up in the REASONATE-2 trial [7]. Second-generation BTK inhibitors such as acalabrutinib and zanubrutinib were also approved for the management of patients with previously untreated and R/R CLL, also with outstanding results [8,9,10]. Venetoclax is the first BCL-2 inhibitor approved for the treatment of patients with CLL, in combination with a BTK inhibitor or a CD20-targeting monoclonal antibody [11,12,13]. BTK has an important role in the activation of the B-cell receptor (BCR)-signaling pathway while the anti-apoptotic BCL-2 protein is overexpressed in tumor cells [14,15]. These two targets shed light on the importance of the tumor microenvironment (TME) of CLL in the natural history of the disease and as a therapeutic target. Indeed, surrounding environmental cells such as mesenchymal stromal cells (MSCs), nurse-like cells (NLCs), and macrophages, and different signaling pathways including the BCR signaling pathway and CD40/CD40L, play an important role in the survival and the proliferation of CLL tumor cells. In this review, we summarize the most important components of TME in CLL and discuss the available data regarding targeted therapy in CLL.

## 2. The Tumor Microenvironment of CLL

The TME plays a growing role in the proliferation and the survival of leukemic cells in patients with CLL. MSCs are among the first components of the TME that were discovered and evaluated by Friedenstein and colleagues [16]. These are self-renewing, non-specialized, and heterogeneous cells with multipotent abilities, and are part of CLL’s protective microenvironment [17]. They express CD73, CD90, CD105, and Cadherin-11, while MSCs should not express hematopoietic markers such as CD34, CD19, CD79a, and CD45, as measured by flow cytometry [18]. It has been demonstrated that MSCs derived from patients with CLL have an increased secretion of transforming growth factor β1 (TGFβ1) in comparison with cells derived from healthy people. In vitro studies had reported that the co-culture of CLL tumor cells and MSCs led to important modifications of gene expression that include an increase in antiapoptotic proteins such as B-cell leukemia/lymphoma-2 (BCL-2), B cell lymphoma-extra-large (BCL-XL), β-catenin, and myeloid leukemia cell differentiation protein 1 (MCL1) [14,19,20,21]. BCL-2 protein is a member of the BCL-2 family, consisting of more than 20 different members that control the permeability of the outer mitochondrial membrane and subsequently play an integral role in apoptosis. The role of BCL-2 in the TME has been investigated. In fact, in vitro studies suggested that the interaction of CLL cells with bone marrow stromal cells, NLCs, and follicular dendritic cells could increase the expression of BCL-2 family pro-survival proteins [22,23]. Moreover, tumor cells in the lymph nodes seem to show a higher expression of anti-apoptotic BCL-2 proteins in comparison with tumor cells in the peripheral blood [24,25]. It has been reported that the co-culture of CLL cells and MSCs provided numerous stimuli of the TME, contributing to the increase in BCL-2 through Notch-1, Notch-2, and Notch-4 signaling, which can partially explain the high levels of the expression of BCL-2 in leukemic cells [14]. These interactions also led to the activation of several pathways, including BCR and TLR [26,27].

BCR consists of an antigen-binding transmembrane immunoglobulin that is specific to antigen and paired with a signal transduction component that contains the CD79a/b heterodimer. It has been shown that deregulated BCR signaling is a driving mechanism for CLL development, progression, and relapse, as there is an increase BCR signaling activity in CLL cells compared to healthy B cells [28]. The CLL cells maintain their important interaction with the TME through the BCR pathway. The BCR is connected to downstream regulators such as phosphoinositide-3-kinase δ (PI3Kδ), BTK, and spleen tyrosine kinase (SYK). The stimulation of BCR by antigen results in the phosphorylation of immunoreceptor tyrosine-based activation motifs (ITAMS) in the cytoplasmic tails of CD79a/b, which contribute to the recruitment and activation of the proximal kinases: Src family tyrosine kinase (LYN) and SYK. After this, BTK is activated, resulting in the activation of phospholipase C2 (PLC 2) and B-cell linker (BLNK), ultimately activating downstream signaling pathways. This includes MAPK/ERK, PI3K/AKT/mTOR, and NF-κB pathways that promote the proliferation, survival, and migration of the malignant clone [29]. The activation of BCR in CLL B cells led to the stimulation of CD40, while stimulation by its ligand activates the BCR upregulation, indicating a crucial interaction between BCR and CD40 in the survival of CLL cells [30]. The CD40 costimulatory molecule is a member of the tumor necrosis factor (TNF) receptor superfamily, and is expressed on various antigen-presenting cells (APCs), as well as some tumor cells. The CD40/CD40L system has a central role in the interaction between different cells of the immune system, as well as interacting with other cell types involved in inflammation. The transduction of the CD40/CD40L signal involves associated proteins such as TRAF and Janus Kinase (JAK) in the cytoplasmic domain of CD40, which are responsible for activating different pathophysiological pathways and involving secondary messengers, such as mitogen-activated protein kinases (MAPKs), PI3K, phospholipase Cγ2 (PLCγ), or a signal transducer and activator of transcription (STAT5). This transduction is responsible for the activation of NF-κB nuclear factor of activated T-cells (NFAT), and the transcription of genes encoding pro-inflammatory cytokines (IL-6, IL-10, TNFα), adhesion (ICAM), or co-stimulation molecules (CD80/B7-1, CD86/B7-2) [31].

Interestingly, aberrant canonical NF-κB activity in CLL cells is promoted through the activation of BCR by MSCs, resulting in survival and proliferation [32]. Moreover, bone marrow MSCs secret several cytokines, such as CXCL12, which is the most studied one, and one which interacts with its specific receptor on leukemic cells, called C-X-C motif chemokine receptor (CXCR4) or CD184 [33]. Mohle and colleagues demonstrated that CLL leukemic cells overexpress CXCR4 in comparison with normal B cells [34]. The activation of CXCR4 stimulates several intracellular pathways, such as PI3k, mitogen-activated protein kinase (MAPK), and a signal transducer and activator of transcription 3 (STAT3) pathways that contributes to the activation of BTK and AKT [15,22,35]. Montresor and colleagues reported that BTK is rapidly activated by CXCL12 in leukemic cells, suggesting that the BCR pathway is interconnected with the CXCL12/CXCR4 axis [15]. Nurse-like cells (NLCs) are myeloid cells found in CLL and represent an important tumor-supporting component of the TME. NLCs can have a dual role, with both tumor-supportive and immunosuppressive actions. In vitro data reported that NLC could protect tumor cells in CLL form through spontaneous or drug-induced apoptosis, endorse migration, and help in the recruitment of tumor-supportive T-cells [22,35,36]. NLCs show a high similarity with tumor-associated macrophages that infiltrate the tissue of lymph node in patients with CLL. They express CD45, CD14, HLA-DR, CD33, and CD68 at different levels, but lack CD106 [37]. NLCs express high levels of stromal-derived factor 1-α (SDF-1α). This chemokine drives CLL cells inside the protective tissue niches through the activation of the CXCR4 receptor expressed on the surface of CLL cells [38]. In addition, this engagement causes the overactivation of both canonical and non-canonical NF-κB pathways through the BCR signaling and the release of BAFF and APRIL, respectively, via antigen-dependent and independent mechanisms [39]. Moreover, NLCs secrete multiple chemokines, such as CXCL12 or CXCL13, that bind to CXCR4 or CXCR5, respectively, on the surface of the CLL cell, attracting it to the TME. In addition, leukemic cells can elaborate chemokines such as CCL3 and CCL4, which bind to CCR1 and CCR3, or CCL17 and CCL22, which bind to CCR4 to recruit T cells and monocytes that can differentiate into NLCs. In addition, the interaction between CD31 on NLCs and CD38 on CLL cells may promote CLL cell survival. NLCs also express BAFF (CD257) and APRIL (CD258), proteins of the TNF family that activate TNF-family receptors on CLL cell-like calcium-modulators and cyclophilin ligand (CAML) interactors, B-cell maturation antigens (BCMAs), or BAFF-receptors and activate NF-κB [40]. In fact, the activation of NF-κB may promote the expression of miR-155, which can enhance BCR signaling and its downstream activation by reducing INPP5D expression, which encodes SHIP1 phosphatase [41]. Furthermore, the activation of NF-κB in CLL cells leads to the production of cytokines such as IL-6, which activates STAT3 in both leukemic and TME cells. Activated leukemic cells lead to the production of high levels of IL-10, which is responsible for the survival of these cells [42].

**Figure 1:**
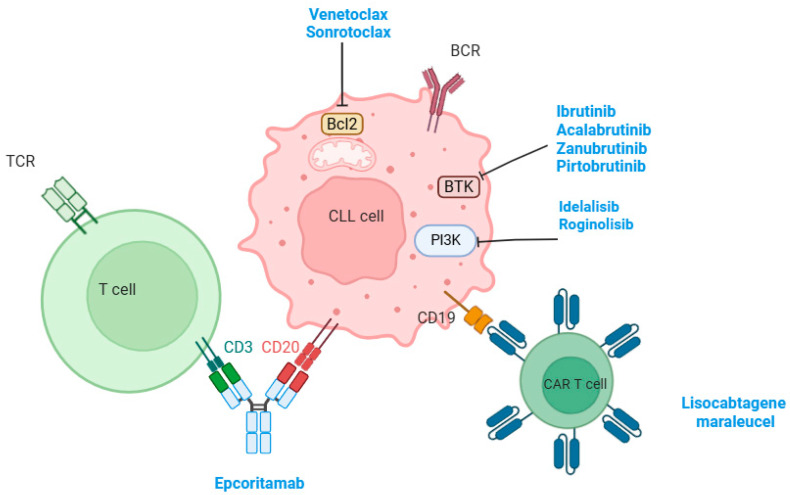
This figure illustrates the different targeted therapies used for the management of CLL and their target on the surface of tumor cells.

The introduction of targeted therapy has dramatically improved the prognosis and outcome of patients with CLL. The first targeted therapy introduced was BTK inhibitors. Ibrutinib is a first-generation BTK inhibitor. It has been shown that ibrutinib restored the proliferation and degranulation of T-cell, enhancing T-cell lytic immune synapse function. It has also been demonstrated that ibrutinib reversed the chronic activation of the T-cell phenotype documented following PD-1 downregulation. Together, this evidence supports the idea that ibrutinib improves the function of T cells [43]. The same phenomenon was seen with acalabrutinib and zanubrutinib (second-generation BTK inhibitors) [44]. The improved function of T cells may also be attributed to distinct effects on CD4+ and CD8+ subsets, most probably related to direct off-target activity of ibrutinib. Ibrutinib inhibits BCR signaling via a direct on-target effect and leads to the redistribution of tumor cells into peripheral blood, which could be considered a disruption of microenvironment interactions [45]. Moreover, Niemann and colleagues demonstrated that patients treated with ibrutinib showed a significant decrease in serum levels of chemokines such as CCL2, CXCL10, CCL3, CCL4, and IL-8, and inflammatory cytokines such IL-10n IL-1ra, TNF-alfa, and IFN-gamma. The authors also found that treatment with ibrutinib disrupts the interaction between tumor-associated macrophages (TAM) and tumor cells in the bone marrow, inhibits the secretion of CXCL13, and decreases the chemoattraction of CLL cells [43]. Moreover, it was demonstrated that ibrutinib blocks BTK and downstream transcription factors within macrophages, leading to the downregulated expression of the chemokines CXCL12 and CXCL13, which could reflect a direct effect of ibrutinib on macrophages [46]. Interestingly, it was reported that treatment with ibrutinib was associated with a reduction in myeloid-derived suppressor cells (MDSC) and the concomitant elevation of classical monocytes after twelve months of treatment with ibrutinib [47]. This effect may be related to the direct effect of BTK inhibition in MDSC, and indirect effects related to reduced tumor burden. Herman and colleagues demonstrated a rapid and sustained downregulation of BCR and NF-κB signaling in CLL cells from both peripheral blood and tissue compartments during ibrutinib treatment, which was associated with decreased tumor proliferation. The authors concluded that ibrutinib inhibits pathways responsible for tumor cell activation and proliferation in vivo with the on-target effect of BTK inhibition [48]. As previously mentioned, BCL-2 is constitutively upregulated in CLL, with an important role in inhibiting apoptotic signals and maintaining the survival of CLL cells [49]. Venetoclax, a selective BCL-2 inhibitor previously named ABT-199, showed an increased capacity to induce apoptosis in primary CLL cells [50]. In vitro studies and reports from healthy human subjects demonstrated that naïve T-cell subsets decreased and memory T-cells increased after receiving venetoclax [51]. De Weerdt and colleagues reported that patients with CLL who received venetoclax plus obinutuzumab, a second-generation CD20-targeting monoclonal antibody, presented decreased numbers of normal T cells, B cells, and NK cells in the peripheral blood. Moreover, the authors documented decreased level of the PD-1 + T-cell phenotype and inflammatory cytokines [52]. It has been reported that TME may play a role in resistance to venetoclax. In fact, the co-stimulation of CD40/CD40L in vitro upregulated other anti-apoptotic proteins, such as MCL-1 and BCL-XL, leading to reduced sensitivity to venetoclax [53]. These findings were confirmed by Elias et al., who found that surviving cells resistant to venetoclax expressed high levels of BCL-XL and/or MCL-1, with a sustained resistance to a second treatment with venetoclax [54]. Figure 1 illustrates the currently available targeted therapies in patients with CLL.

## 3. Targeted Therapy for the Management of CLL

### 3.1. BTK Inhibitors

As previously described, BTK plays a crucial role in BCR signaling. The advent of small-molecule BTK inhibitors shifted the treatment paradigm of patients with CLL during the last decade. Ibrutinib, formerly named PCI-32675, was the first oral covalent BTK inhibitor approved by the Food and Drug Administration (FDA) for the management of patients with CLL. Initially, ibrutinib was approved for patients with R/R CLL in February 2014 based on the results of a phase I–II study with an ORR of 71%. The 26-month PFS rate was 75% while the overall survival (OS) rate was 83% [6]. Ibrutinib was then approved as first-line therapy for CLL in March 2016 based on the results of the phase III REASONATE-2 trial. This trial randomized older patients to receive continuous ibrutinib or 12 cycles of chlorambucil. The study met its primary endpoint of PFS, which was not reached in the ibrutinib arm, while it was reached at 18.9 months in the control arm (Hazard ratio (HR), 0.16; *p* < 0.001). Moreover, ibrutinib was associated with significantly longer OS [7]. At a median follow-up of 7.4 years, 42% of patients (57/136) in the ibrutinib arm remained on treatment, and the estimated 7-year PFS rate was 59% [55]. In the ILLUMINATE phase III trial, older patients with previously untreated CLL were randomized to receive either continuous ibrutinib plus six cycles of obinutuzumab, a second-generation CD20 monoclonal antibody, or six cycles of chlorambucil and obinutuzumab, in contrast with the REASONATE-2 trial where patients did not receive the CD20 monoclonal antibody. Once again, the study met its primary endpoint of PFS, which was not reached in the experimental arm and reached at 19.0 months in the control arm (HR, 0.23; *p* < 0.0001). Moreover, the 30-month PFS rate was 79% in the control arm and 31% in the experimental arm [56]. Furthermore, at a median follow-up of 45 months, the median PFS was not reached in the experimental arm. Interestingly, in the ILLUMINATE trial, the proportion of patients with undetectable minimal residual disease (uMRD), defined as less than 0.01% by flow cytometry, was 38% of patients, in comparison with rare u-MRD remission with ibrutinib monotherapy [57]. Unfortunately, there are no randomized data comparing single-agent ibrutinib and ibrutinib with obinutuzumab. In clinical practice, ibrutinib is used as a monotherapy. The Alliance A041202 is another phase III trial in older patients with previously untreated CLL. Patients were randomly assigned to receive six cycles of bendamustine plus rituximab, continuous ibrutinib, or continuous ibrutinib with six cycles of rituximab. Both ibrutinib arms had significantly longer PFS in comparison with the bendamustine plus rituximab arm (HR, 0.39 with ibrutinib alone; HR, 0.38 with ibrutinib and rituximab; *p* < 0.001). The PFS benefit was observed in the subgroup of patients with IGHV-unmutated CLL, and no difference was seen among patients with IGHV-mutated CLL. Interestingly, there was no difference in terms of PFS between the two ibrutinib arms, showing that the addition of rituximab did not improve outcomes in patients treated with ibrutinib [58,59]. The ECOG E1912 was the first phase III trial that randomized patients younger than 70 years with previously untreated CLL who were eligible to receive chemotherapy. This trial compared continuous ibrutinib plus six cycles of rituximab with six cycles of chemoimmunotherapy with fludarabine, cyclophosphamide, and rituximab (FCR). The trial met its primary endpoint of PFS in the intention-to-treat (ITT) population (HR, 0.35; *p* < 0.001). An improvement in PFS was seen in groups of both IGHV-unmutated CLL (HR, 0.27; *p* < 0.001) and IGHV-mutated CLL (HR, 0.27; *p* < 0.001) at a median follow-up of 5.8 years. Moreover, ibrutinib plus rituximab was associated with statistically longer OS in comparison with FCR (HR, 0.47; *p* = 0.018). This improvement was seen specifically in patients with unmutated IGHV, but not for patients with IGHV-mutated CLL [60,61]. The FLAIR is another phase III trial with the same design as the E1912 trial, randomizing patients to receive either six cycles of FCR or continuous ibrutinib and six cycles of rituximab. The study also met its primary endpoint of PFS with an HR of 0.44 (*p* < 0.0001). At a median follow-up of 53 months, the median PFS was not reached in the ibrutinib arm, while it was 67 months in the FCR arm. The advantage of PFS of ibrutinib plus rituximab was only seen among patients with unmutated IGHV. Moreover, there was no OS benefit in the analysis [62]. Second-generation BTK inhibitors were subsequently developed with more selectivity for the inhibition of BTK. Acalabrutinib, formerly known as ACP-196, is a second-generation, highly selective, potent, and irreversible BTK inhibitor. It has been evaluated in a phase I-II trial as a monotherapy in patients with R/R CLL, including those with del(17p). The ORR was 95% at a median follow-up of 14.3 months. Interestingly, the ORR was 100% among patients with del(17p) [8]. The ASCEND phase III trial randomized patients to receive either acalabrutinib as monotherapy or investigator’s choice (rituximab plus idelalisib or rituximab plus bendamustine) in patients with R/R CLL, including those with del(17p). The primary endpoint of PFS in the ITT population was met in the trial. It was not reached in the acalabrutinib arm and reached at 16.5 months in the control arm (HR, 0.31; *p* < 0.0001) [63]. At a median follow-up of 46.5 months, the median OS was not reached in both arms. The 42-month PFS and OS were 62% and 78%, respectively, in the acalabrutinib arm versus 19% and 65%, respectively, in the control arm. Acalabrutinib was superior to the control arm in all subgroups, particularly in patients with del(17p) and TP53 mutations [64]. Acalabrutinib was also evaluated in patients with previously untreated CLL, with or without obinutuzumab, and compared with treatment using chlorambucil plus obinutuzumab, in the ELEVATE TN phase III trial. The study included only 25% of patients with del(17p) or TP53 mutations. Acalabrutinib, as monotherapy or in combination with obinutuzumab, was associated with significantly longer PFS in comparison with chlorambucil plus obinutuzumab. Median PFS was not reached in both arms of acalabrutinib, and was 22.6 months in the control arm (HR, 0.1; *p* < 0.0001 with the combination of acalabrutinib; HR, 0.2; *p* < 0.0001 with acalabrutinib alone) [9]. At a median follow-up of 74.5 months, the estimated 72-month PFS rate was 78% for the acalabrutinib and obinutuzumab arm, 62% for the acalabrutinib monotherapy arm, and 17% for the chlorambucil and obinutuzumab arm. The median PFS for the 79 patients who crossed from the control arm to acalabrutinib alone was not reached when treated with acalabrutinib. Interestingly, acalabrutinib plus obinutuzumab was associated with a statistically significant improvement in PFS in comparison with acalabrutinib alone (HR, 0.58; *p* = 0.02) [65]. Zanubrutinib, another highly potent and selective second-generation BTK inhibitor, was evaluated in a first-line setting in the SEQUOIA phase III in comparison with rituximab and bendamustine. We should note that patients with del(17p) were excluded from the randomized cohort. Zanubrutinib was associated with significantly longer PFS than bendamustine plus rituximab (HR, 0.42; *p* < 0.0001) [10]. At a median follow-up of 61.2 months, the median PFS was not reached with zanubrutinib but was reached at 44.1 months in the control arm (HR, 0.29; *p* = 0.0001). The improvement in PFS was seen in both mutated and unmutated IGHV groups [66]. Arm C of the SEQUOIA trial evaluated zanubrutinib as monotherapy in patients with previously untreated CLL with del(17p). The ORR was 94.5% at a median follow-up of 18.2 months. The 18-month PFS rate was 88.6% [67]. In patients with R/R CLL, zanubrutinib was directly compared with ibrutinib in the ALPINE phase III trial. Zanubrutinib was associated with a significantly longer PFS than ibrutinib (HR, 0.65; *p* = 0.002) [68]. Interestingly, the improvement in PFS with zanubrutinib was observed across major subgroups, notably in patients with del(17p) or TP53 mutations (HR, 0.52; *p* = 0.005). The ORR was higher with zanubrutinib in comparison with ibrutinib (85.0% versus 74.8%, *p* = 0.001). Zanubrutinib was also associated with fewer adverse events than ibrutinib [68]. Acalabrutinib was also compared with ibrutinib in the ELEVATE-RR non-inferiority phase III trial in patients with R/R CLL, including patients with del(11q) or del(17p). At a median follow-up of 40.9 months, acalabrutinib demonstrated non-inferior PFS in comparison with ibrutinib (HR, 1.00), with lower cardiovascular AEs [69]. Given the results of the ELEVATE-RR and ALPINE phase III trials, second-generation BTK inhibitors became the preferred BTK inhibitors for the treatment of CLL.

Pirtobrutinib is a selective, non-covalent (reversible) BTK inhibitor and is active in both wild-type BTK and cysteine 481-mutant BTK. BTK C481 mutation is the most frequent mutation found in patients who developed resistance to covalent BTK inhibitors such as ibrutinib, acalabrutinib, and zanubrutinib [70]. Pirtobrutinib was evaluated in the BRUIN phase I-II trial in patients with R/R B-cell malignancies. Pirtobrutinib was effective in patients previously treated with a covalent BTK inhibitor. Overall, 247 patients with R/R CLL were treated with pirtobrutinib. All patients previously received a covalent BTK inhibitor (77% of them stopped it due to disease progression while 23% discontinued treatment due to toxicity or other reasons), and 41% of them were previously exposed to venetoclax. The median number of previous lines was three, and 47% of patients presented with del(17p) or TP53 mutations, while 85% of patients had unmutated IGHV disease. The ORR was 82.2% and the median PFS was 19.6 months. Interestingly, the ORR among patients who previously failed venetoclax was 79%, with a median PFS of 16.8 months [71]. Based on these exciting results, the FDA approved pirtobrutinib for the management of patients with R/R CLL after at least two previous treatment lines, including a covalent BTK inhibitor and BCL-2 inhibitor.

The BRUIN CLL-321 is the first randomized phase III comparing pirtobrutinib with idelalisib plus rituximab or bendamustine plus rituximab in patients with R/R CLL previously treated with a BTK inhibitor. A total of 238 patients were enrolled. The primary endpoint of PFS was met at a median follow-up of 11.6 months, with an HR of 0.55, *p* = 0.0007. The efficacy of pirtobrutinib was observed across all subgroups, including patients previously treated with venetoclax (HR, 0.54), patients with complex karyotype (HR, 0.34), and patients with del(17p) and/or TP53 mutation (HR, 0.52). Pirtobrutinib was associated with a favorable safety profile [72]. Table 1 summarizes the major phase III trials evaluating BTK inhibitors alone or in combination with CD20-targeting monoclonal antibodies.

### 3.2. BCL2 Inhibitors

Venetoclax, formerly named ABT-199, is a selective BCL-2 inhibitor that is implicated in the apoptosis of CLL cells in vitro and in xenograft models as previously mentioned [45]. Venetoclax in combination with Obinutuzumab was evaluated in first-line setting in elderly patients in the phase III CLL14 trial. Patients randomized to receive six cycles of Obinutuzumab combined with either 12 cycles of venetoclax or chlorambucil. The study met its primary endpoint of PFS with an HR of 0.35 (*p* < 0.001). The benefit of venetoclax and obinutuzumab was observed across all subgroups including del(17p) or TP53-mutated subgroups [11]. At a median follow-up of 76.4 months, the median PFS was 76.2 in the venetoclax arm compared to 36.4 months in those without (HR, 0.40; *p* < 0.0001). The study did not show a statistical improvement in OS (6-year OS rate of 78.7% in the venetoclax arm versus 69.2% in the control arm (HR, 0.69; *p* = 0.052). The authors demonstrated that u-MRD at 12 months after the end of treatment is predictive of longer PFS and OS [74]. CLL13 is another phase III trial in patients with previously untreated CLL, where patients were randomized to receive either venetoclax plus rituximab, venetoclax plus obinutuzumab, venetoclax plus obinutuzumab and ibrutinib, or chemoimmunotherapy. The chemoimmunotherapy regimens consisted of FCR in patients 65 years old and younger, or bendamustine plus rituximab in patients older than 65 years. Interestingly, patients with del(17p) and TP53 mutations were excluded from the study. The study showed that venetoclax in combination with obinutuzumab, with or without ibrutinib, was associated with higher rates of u-MRD (86.5%, and 92.2%, respectively) than the chemoimmunotherapy group (52.0%), *p* < 0.001 [75]. At a median follow-up of 50.7 months, the median PFS was significantly longer in the venetoclax with obinutuzumab with or without ibrutinib (NR in both arms) in comparison with the chemoimmunotherapy group (59.4 months), with an HR of 0.47 (*p* < 0.001) for venetoclax and obinutuzumab, and an HR of 0.30 (*p* < 0.001) for venetoclax with obinutuzumab and ibrutinib. Moreover, the median was also statistically higher with venetoclax and obinutuzumab, with or without ibrutinib (NR in both arms), in comparison with venetoclax and rituximab (63.2 months), with an HR of 0.57 and 0.38 for venetoclax plus obinutuzumab and venetoclax plus obinutuzumab and ibrutinib, respectively [76].

Venetoclax was also evaluated in patients with R/R CLL in the MURANO phase III trial. Patients were randomized to receive either six cycles of bendamustine and rituximab or a 24-month fixed duration of venetoclax plus rituximab. The trial met its primary endpoint of PFS. The combination of venetoclax and rituximab was associated with significantly higher 24-month PFS rate in comparison with the control group of bendamustine and rituximab (84.9% versus 36.3%; HR, 0.17; *p* < 0.001). Moreover, venetoclax and rituximab were associated with significantly longer OS. Interestingly, patients with IGHV-unmutated CLL has significantly lower PFS in comparison with patients with IGHV-mutated CLL among patients treated with venetoclax and rituximab [77].

Sonrotoclax is a novel, potent, and selective BCL-2 that was found to be effective in both BCL-2 wild-type and BCL-2 mutations. It should be noted that several BCL-2 mutations were observed in patients treated with venetoclax. BCL-2 G101V is an acquired mutation reported in nearly half of patients who were treated and failed to improve with venetoclax, and was not present at the beginning of the treatment [78]. Other BCL-2 mutations were also reported, such as D103Y, A113G, V156D, and R129L, in patients who progressed after treatment with venetoclax. Sonrotoclax has shown potent antitumor activity against wild-type BCL-2, BCL-2 G101V-mutated, and BCL-2 D103Y-mutated xenograft models [79]. The BGB-11417-204 is a phase II trial comparing sonrotoclax with zanubrutinib and zanubrutinib alone in patients with previously untreated CLL (NCT06637501).

### 3.3. Combination of BTK and BCL2 Inhibitors

Another strategy that has been evaluated for the management of patients with CLL is the combination of BTK and BCL-2 inhibitors in a fully oral, time-limited regimen. The combination of ibrutinib and venetoclax is the most studied regimen. The first report concerning the association of ibrutinib and venetoclax came from the MD Anderson Cancer Center in patient with previously untreated CLL. Patients were treated with 24 cycles with the addition of twelve other cycles in case of persistent bone marrow MRD with a sensitivity of 10^−4^. The majority of enrolled patients (92%) had high-risk disease such as unmutated IGHV, TP53 aberration, or chromosome 11q deletion. At the end of 12 cycles of combined treatment, 88% of patients achieved CR or CR with incomplete count recovery. Moreover, 61% had remission with undetectable MRD [12]. At a median follow-up of 38.5 months, the 36-month PFS rate was 93% while the 36-month OS rate was 96% [80].

The randomized phase II CAPTIVATE trial enrolled patients with previously untreated CLL and who received treatment with a fixed-duration schedule of ibrutinib and venetoclax (12 cycles) or MRD-driven schedule consisting of adding more cycles of the combination depending on the MRD results. In both cohorts, MRD was undetectable in approximately 75%. Patients treated with a fixed-duration schedule had a PFS rate of 70% at 4.5 years [81]. The UK FLAIR randomized phase III trial compared the combination of ibrutinib plus venetoclax with FCR in patients with previously untreated CLL. Patients with del(17p) were excluded. The experimental arm was guided by the MRD and the duration varied from two to six years. The combination of ibrutinib and venetoclax was associated with a 3-year PFS rate of 97.2%, while 76.8% was reported for the control group. with an HR of 0.13, *p* < 0.001. Moreover, ibrutinib and venetoclax are associated with a significantly higher OS in comparison with FCR with an HR of 0.31, *p* < 0.005. Interestingly, the advantage of OS was not seen among patients with mutated IGHV [13]. The GLOW phase III trial is another trial comparing ibrutinib with venetoclax to obinutuzumab and chlorambucil in elderly patients or younger patients with comorbidities unfit for FCR. Once again, patients with del(17p) were excluded. The experimental arm was associated with a significantly longer PFS than the control arm (HR, 0.21; *p* < 0.001) [82,83]. The combination of ibrutinib and venetoclax was approved in the European Union based on these results, but not by the FDA.

The DFCI phase II trial evaluated the combination of acalabrutinib, venetoclax, and obinutuzumab in patients with previously untreated CLL in a fixed-duration regimen for 25 cycles. At cycle 16, 38% of patients had CR with undetectable MRD in bone marrow [84]. Davids and colleagues recently reported another phase II of patients with previously untreated CLL, evaluating acalabrutinib with venetoclax and obinutuzumab. The study enrolled 72 patients, including 45 patients with TP53 aberration. The CR with undetectable MRD rates in bone marrow at the start of cycle 16 were 42% in patients with TP53 aberration and 42% in all-comers. The undetectable MRD rates in bone marrow were 71% and 78%, respectively. The 48-month PFS rates were 70% and 88% in patients with and without TP53 aberrations, while the 48-month OS rates were 96% and 100%, respectively [85]. The results of the AMPLIFY (ACE-CL-311) randomized phase III were recently presented at the 2024 American Society of Hematology annual meeting. The study randomized treatment-naïve patients with CLL without del(17p) or TP53 mutation to receive either acalabrutinib plus venetoclax, acalabrutinib plus venetoclax plus obinutuzumab, or chemoimmunotherapy (FCR or bendamustine plus rituximab). The primary endpoint was median PFS in acalabrutinib plus venetoclax in comparison with chemoimmunotherapy. The study met its primary endpoint, and the experimental arm was associated with longer PFS than chemoimmunotherapy (HR, 0.65; *p* = 0.0038). The median PFS was not reached in both arms of acalabrutinib, while it was reached at 47.6 months in the control group. Moreover, the association of acalabrutinib, venetoclax, and obinutuzumab was associated with a statistical improvement in PFS in comparison with chemo-immunotherapy (HR, 0.42; *p* < 0.0001) [86].

Zanubrutinib was also evaluated in combination with venetoclax in patients with previously untreated CLL with del(17p) and/or TP53 mutation in the SEQUOIA trial arm D. Patients were 65 years old or between 18 and 64 years with comorbidities. The preliminary results were presented at the 2024 European Hematology association meeting, showing promising efficacy. A total of 66 patients were enrolled, and the ORR was 100% among 65 response-evaluable patients, including 45% of CR + CR with incomplete recovery. The median PFS was not reached and the 36-month PFS rate was 92% [87]. The BOVeb phase II trial evaluated the combination of zanubrutinib with obinutuzumab and venetoclax in patients with previously untreated CLL. A total of 39 patients were treated, of whom 72% presented with IGHV-unmutated CLL and 13% had del(17p) or TP53 mutation. At a median follow-up of 25.8 months, 89% of patients (33/37) presented with undetectable MRD [88].

The combination of sonrotoclax and zanubrutinib was under evaluation in the ongoing BGB-11417-101 phase I trial in patients with previously untreated CLL. The preliminary results were presented at the 2024 ASH meeting. The combination was associated with a manageable safety profile and very encouraging efficacy, with an ORR of 100% in the 108/112 evaluable patients at a median follow-up of 18.3 months [89]. Cheah and colleagues reported the updated results of the cohort of patients with R/R CLL at the 2025 European Hematology Association congress. Overall, forty-seven patients with R/R CLL/SLL received the combination of zanubrutinib and sonrotoclax. At a median follow-up of 29.4 months, the ORR was 95.7% including 50% CR. Only two PFS events occurred during follow-up and the 24-month PFS rate was 94.5%, with a tolerable safety profile [90]. The BGB-11417-301 registrational phase III trial is currently recruiting patients with previously untreated CLL and compared with venetoclax and obinutuzumab. Pirtobrutinib was also evaluated in combination with venetoclax with or without rituximab in a fixed-duration regimen in patients with R/R CLL in the BRUIN phase I trial. Nearly two thirds of patients had already received a covalent BTK inhibitor. The combination was associated with promising efficacy. The ORR was 93.3% in the pirtobrutinib and venetoclax arm, and 100% with pirtobrutinib plus venetoclax plus rituximab. Interestingly, 87.5% and 90.0% of patients achieved u-MRD after 12 cycles of treatment in the pirtobrutinib plus venetoclax and pirtobrutinib plus venetocalx plus rituximab groups, respectively. Moreover, the 18-month PFS rate was 92.9% in the pirtobrutinib plus venetoclax group, and 80.0% in the pirtobrutinib plus venetoclax plus rituximab group [91]. Table 2 shows the results of the major phase III trial evaluating the combination of BTK inhibitors and BCL-2 inhibitors.

### 3.4. PI3K Inhibitors

Idelalisib, formerly named GS-1101 and CAL-101, is a potent, oral, selective inhibitor of PI3K. It was evaluated as monotherapy in a phase I trial in patients with heavily pretreated R/R CLL. A total of 54 patients received idelalisib; 91% of patients had unmutated IGHV and 24% of patients had del(17p) or TP53 mutation. ORR was observed in 56% of patients (30/54), the median PFS was 17 months, and the median DOR was 18 months, with no dose-limiting toxicities observed [92]. Idelalisib was then evaluated in a phase III trial in combination with rituximab compared to rituximab with placebo, and the primary endpoint was the PFS. The authors found that idelalisib and rituximab were associated with a significantly higher PFS in comparison with rituximab and placebo (NR versus 5.5 months, respectively; HR, 0.15; *p* < 0.001). The 12-month OS rate was 92% in the idelalisib arm and 80% in the placebo arm (HR, 0.28; *p* = 0.02) [93]. Roginolisib (IOA-244), a novel PI3K delta inhibitor, is currently under evaluation in combination with venetoclax and rituximab in patients with R/R CLL (NCT06644183).

### 3.5. Chimeric Antigen Receptor T-Cell Therapy

The development of chimeric antigen receptor (CAR) T-cell therapy has dramatically improved the management of patients with hematological malignancies. CAR T-cells are a synthetic product developed from an antibody sequence to bind target cell surface antigens with high specificity via a single-chain variable fragment (scFv) recognition domain [94]. The scFv domain is linked to an intracellular signaling domain derived from the CD3 zeta (CD3ζ) chain of the endogenous T-cell receptor (TCR) to stimulate the activation of T cells after antigen binding through an extracellular hinge domain and a transmembrane domain. The product is known as the first-generation CAR T-cell [95]. Multiple efforts were made thereafter to improve CAR T-cells and contribute to the production of second- and third-generation CAR T-cells by adding costimulatory signaling endodomains such as CD28 and CD137 (also known as 4-1BB) that were located between the transmembrane and CD3 signaling domains [96,97]. CD19-targeting and BCMA-targeting CAR T-cells were approved for the management of several hematological malignancies. Axicabtagene ciloleucel (Axi-cel), tisangelecleucel (tisa-cel), and lisocabtagene maraleucel (liso-cel) are CD19-targeting CAR T-cells approved for the treatment of patients with R/R large B cell lymphoma (LBCL) [98]. Brexucabtagene autoleucel (brexu-cel) is another CD19-targeting CAR T-cell approved for the management of R/R B-cell acute lymphoblastic leukemia and R/R mantle cell lymphoma (MCL) [99,100]. Liso cel is also approved for the management of patients with R/R follicular lymphoma and MCL [101,102]. CAR T-cell was also evaluated in patients with R/R CLL. Tisa-cel was evaluated in patients with R/R CLL. In one report of 14 patients, tisa-cel was associated with an ORR of 57%, including 28% CR, a median PFS of 7 months, and a median OS of 29 months [103]. In another report of 38 patients, Frey and colleagues reported an ORR of 44% including 28% CR with tisa-cel, a median PFS of 1.8 months, and a median OS of 64 months [104]. Liso-cel is the first CAR T-cell to be approved by the FDA in March 2024 for the management of patients with R/R CLL who failed at least two previous lines of treatment, including a BTK inhibitor and a BCL-2 inhibitor, based on the results of the TRANSCEND CLL 004 phase I/II trial. This study is the largest prospective study to date to include patients with R/R CLL. A total of 117 patients, with a median previous treatment line of 5, were infused. All infused patients had already received a BTK inhibitor, while 60% (70/117 patients) had already failed venetoclax. The rate of CR (including patients with incomplete marrow recovery) was 18%, with an ORR of 47%. Interestingly, 64% of patients achieved MRD negativity with a sensitivity of 10^−4^ in the peripheral blood. The median PFS was 18.0 months. Regarding safety, 9% of patients (10/117) presented with grade 3 cytokine release syndrome (CRS) (with no grade 4 or 5 events), while 18% of patients (21/117) presented with grade 3 neurotoxicity, and 1% (1/117) grade 4 neurotoxicity [105]. In TRANSCEND CLL 004, liso-cel was also evaluated in combination with ibrutinib in another cohort of heavily pretreated patients. All patients had already been treated with ibrutinib, and 98% of patients had high-risk cytogenetics, including TP53 mutation, del(17p), and unmutated IGHV. The combination was associated with a promising efficacy, with an ORR of 86% and a median DOR of 41.4 months. Surprisingly, the median PFS was 31.4 months while the median OS was not reached. Furthermore, 86% of patients achieved u-MRD in blood. The safety profile was manageable, with no new safety alerts. The combination of liso-cel with ibrutinib could be an interesting new therapeutic option in patients with heavily pretreated CLL [106]. Another phase II trial evaluated huCART-19, a CD19-targeting CAR T-cell, in combination with ibrutinib in patients with R/R CLL. A total of 19 patients were infused. The 48-month PFS and OS rates were 70% and 84%, respectively. The safety profile was acceptable and no new concerns were reported [107]. Rapcabtagene autoleucel (YTB323), is a novel autologous CD19-targeting CAR T-cell, developed using a next-generation platform in less than two days. The preliminary data showed encouraging efficacy in patients with R/R DLBCL with an ORR of 75% to 80% with a favorable safety profile [108]. The rapcabtagene autoleucel will be investigated in a phase I-II trial in combination with ibrutinib in patients with R/R CLL/SLL and is a monotherapy in patients with LBCL or ALL (NCT03960840). JCAR014 is another autologous CD19-targeting CAR T-cell that was evaluated in a phase I-II trial and was associated with an ORR of 71%, including 17% CR. The median PFS was 8.9 months, while the median OS was 25 months [109]. Obecabtagene autoleucel (formerly known as AUTO1) is a fast off-rate autologous CD19-targeting CAR T-cell that was evaluated in the ALLCAR19 phase Ib-II trial. It has been associated with impressive results and a median OS of 15.6 months in patients R/R ALL, a 12-month OS rate of 61.1%, and a very good safety profile [110]. In the CLL cohort, 80% of patients (4/5 patients) achieved flow-negative remission in the bone marrow [111]. A phase I-II trial, sponsored by the National Cancer Institute, is currently evaluating a fully human CD19-targeting CAR T-cell (Hu19-CD828Z) in patients with CLL/SLL (NCT06364423). There are several ongoing trials evaluating CAR T-cells in patients with R/R CLL. LMY-920 is a BAFF-ligand CAR T-cell that is currently under evaluation in patients with R/R CLL in a phase I trial (NCT06916767). In fact, preclinical data suggest that BAFF-R CAR T-cells have shown anti-tumor activity in patients with non-Hodgkin lymphoma, including patients with R/R CLL [112,113].

### 3.6. Bispecific Antibodies

Bispecific antibodies (BsAbs) and bispecific T-cell engagers (BiTEs) are a new class of promising therapeutic strategies for B-cell malignancies. Blinatumomab, a BiTE-targeting CD3 on T cells and CD19 on B cells, has shown preclinical efficacy in CLL by inducing the autologous T-cell killing of CLL cells and exhibiting cytotoxicity against CLL cells in both R/R and treatment-naïve CLL cell lines [114]. However, clinical data on blinatumomab in CLL are very limited. The combination of blinatumomab and lenalidomide is under investigation in a phase I trial in patients with relapsed NHL, including R/R CLL/SLL (NCT02568553). AZD0486 is a fully human BiTE also targeting CD19, and CD3 is currently under evaluation in the Soundtrack-E phase I/II trial, either as a monotherapy or in combination with acalabrutinib in patients with R/R CLL/SLL (NCT06564038). Epcoritamab, a BsAb targeting CD3 on T cells and CD20 on B cells, is approved for the treatment of patients with R/R diffuse large-B cell lymphoma and follicular lymphoma. Preclinical data has demonstrated that epcoritamab is associated with significant in vitro cytotoxic activity against CLL cells derived from patients with treatment-naïve CLL or pretreated with a BTK inhibitor. Interestingly, this activity was independent of the expression of CD20 on CLL cells [115]. Epcoritamab is currently under evaluation in patients with R/R CLL and Richter transformation in the EPCORE CLL-1 phase I/II trial, and was associated with promising efficacy. Overall, 40 patients were treated: 23 patients in the expansion cohort and 17 patients in the optimization cohort. All patients were previously treated with a BTK inhibitor, 88% were also treated with chemoimmunotherapy, and 85% were previously exposed to a BTK inhibitor and BCL-2 inhibitor. In the expansion cohort, the ORR was 61%, including 39% CR. The median PFS was 12.8 months and the median OS was not reached. The 15-month OS rate was 65%. Epcoritamab was also effective in patients with TP53 aberrations (63% of the whole cohort) with an ORR of 67%, including 33% of CR. The safety profile was consistent with previously reported adverse events with epcoritamab (96% of CRS in the expansion cohort, including 17% of grade 3. No neurologic events were reported [116]. The combination of venetoclax and epcoritamab is under evaluation in patients with R/R CLL/SLL in the (AETHER) phase I/II trial (NCT05791409). In fact, preclinical data has shown that epcoritamab in combination with venetoclax is associated with increased killing of CLL cells in comparison with epcoritamab or venetoclax alone, especially in samples from patients who progressed while receiving a BTK inhibitor [115]. Moreover, NVG-111 is a BiTE-targeting Receptor tyrosine kinase-like Orphan Receptor 1 (ROR1)—a protein which is expressed at high levels on many types of cancers, including CLL and CD3—and was evaluated in a phase I trial in patients with R/R ROR1 + malignancies including patients with CLL (NCT04763083). The preliminary results showed that NVG-111 is associated with an ORR of 55% (6/11 patients), including three patients with CLL who achieved undetectable MRD in peripheral blood [117]. JNJ-80948543, a trispecific antibody targeting CD79b, CD20, and CD3, is currently under investigation in patients with NHL and CLL (NCT05424822).

## 4. Challenges of CLL-Directed Treatments

BTK inhibitor and BCL-2 inhibitors changed the treatment paradigm of patients with CLL with long-term remission. However, many patients relapse, with the emergence of mutations. In patients treated with a covalent BTK inhibitor, Woyach and colleagues reported the emergence of BTK and PLCG2 mutations [118]. Moreover, recurrent G101V BCL-2 mutation was reported in patients with CLL resistant to venetoclax [78]. The discovery of these mutations may help in selecting BTK and BCL-2 inhibitors. However, it is known that not all patients will present resistance mutations at the time of relapse, suggesting that relapse could be related to alternative mechanisms of resistance. Another issue is the presence of multiple strategies in the first-line setting, with few head-to-head comparisons. Table 3 shows the major ongoing phase III trials in previously untreated or R/R CLL that evaluate new drugs and new strategies for the management of patients with CLL. Richer transformation, commonly to diffuse large B-cell lymphoma, is reported in 5% to 10% of patients with CLL, which remains an unmet medical need with a median OS of less than one year [119]. It seems that covalent and non-covalent BTK inhibitors and venetoclax have shown activity in patients with Richter transformation [120,121].

## 5. Conclusions

The management of CLL has been fundamentally transformed over the past decade by the introduction of targeted therapies. However, the TME remains a critical factor in CLL pathogenesis and therapeutic resistance, with stromal and nurse-like cells supporting leukemic cell survival and contributing to drug resistance. An improved understanding of these interactions is paving the way for innovative combination strategies and the development of new molecular targets.

BTK inhibitors and BCL-2 inhibitors remain the most active classes of drugs, with superior outcomes in comparison chemoimmunotherapy. Ongoing trials are trying to establish the best strategy for long-term outcomes (sequential treatment, or a concurrent doublet combination with BTK and BCL-2 inhibitors or triplet combination with CD20-targeting monoclonal antibodies).

Non-covalent BTK inhibitors such as pirtobrutinib are emerging, as well as T cell-directed therapies, such as CAR T-cells and bispecific antibodies.

## Figures and Tables

**Table 1:** Major phase III trial of a bruton tyrosine kinase inhibitor, alone or in combination with CD20 monoclonal antibodies.

Trial	Treatment Arms	Line of Treatment	Population	Nb of Pts	Median Age (y) (Range)	Disease Characteristics	Primary Endpoint	U-MRD Rate (PB or BM)
REASONATE-2 [7,55]	Ibru vs. Clb	1	65 years or older	269	73 (65–89) vs. 72 (65–90)	U-IGHV: 43% vs. 45% Del(11q): 21% vs. 19% Del(17p) excluded	mPFS: 8.9 y vs. 1.3 y	NA
ILLUMINATE [56,57]	Ibru + Obn vs. Clb + Obn	1	65 y or older younger than 65 y with comorbidities	229	70 (66–75) vs. 72 (66–77)	U-IGHV: 62% vs. 53% Del(17p): 12% vs. 16% TP53 mutation: 12% vs. 15% Del(11q): 12% vs. 19%	mPFS: NR vs. 22 months	38% vs. 25% (*p* = 0.033)
Alliance A041202 [58,59]	Benda + R vs. Ibru vs. Ibru + R	1	65 y or older	547	71 (65–89)	U-IGHV: 58% vs. 63% vs. 61% Del(17p): 8% vs. 5% vs. 6% TP53 mutation: 9% vs. 9% vs. 12% Del(11q): 18% vs. 19% vs. 21%	mPFS: 44 m vs. NR vs. NR 48-month PFS rate: 47% vs. 76% vs. 76%	4% vs. 0.3% vs. 2%
ECOG E1912 [60,61]	Ibru + R vs. FCR	1	70 years or younger	529	57 ± 7.4 (mean)	U-IGHV: 75% vs. 62% Del(11q): 22% vs. 22% Del(17p) excluded	36-month PFS rate: 89.4% vs. 72.9% (*p* < 0.001)	At cycle 12: 8.3% vs. 59.2%
FLAIR [62]	Ibru + R vs. FCR	1	Between 18 and 70 y	771	62 y	U-IGHV: 50% vs. 50% Del(17p) excluded	mPFS: NR vs. 67 m (*p* < 0.0001) 48-month PFS rate: 85.6% vs. 73.0%	NA
ASCEND [63,64]	Acala vs. R + idela or R + benda	R/R	Pts aged 18 years and higher	310	68 y (32–89)	U-IGHV: 77% vs. 82% Del(17p) : 18% vs. 14% Del(11q): 25% vs. 29% TP53 mutation: 26% vs. 22%	MPs: NR vs. 16.5 m 42-month PFS rate: 62% vs. 19%	NA
ELEVATE TN [9,65]	Acala + Obn vs. Acala vs. Clb + Obn	1	65 y or older younger than 65 y with comorbidities	535	70 y	U-IGHV: 58% vs. 67% vs. 66% Del(17p): 9% vs. 9% vs. 9% Del(11q): 17% vs. 17% vs. 19% TP53 mutation: 12% vs. 11% vs. 12%	mPFS: NR vs. NR vs. 22.6 m 72-month PFS rate: 78% vs. 62% vs. 17%	13% (acala + Obn) vs. 8% (Clb + Obn) with CR or CRi
SEQUOIA [10,66]	Zanu vs. R + benda	1	65 y or older younger than 65 y with comorbidities	590	70 (66–75)	U-IGHV: 53% vs. 52% Del(11q): 18% vs. 19% TP53 mutation: 6% vs. 6% Del(17p) excluded	mPFS: NR vs. 44.1 m	NA
ALPINE [68,73]	Zanu vs. Ibru	R/R CLL	Pts aged 18 years and higher	652	67 y (35–90) vs. 68 (35–89)	U-IGHV: 73% vs. 73% Del(11q): 28% vs. 27% Del(17p): 14% vs. 15% TP53 mutation: 9% vs. 8%	mPFS: NR vs. 34.2 m *p* = 0.002 36-month PFS rate: 65% vs. 54%	NA
ELEVATE RR [69]	Acala vs. Ibru	R/R CLL	Pts aged 18 years and higher	533	66 (41–89) vs. 65 (28–88)	U-IGHV: 82% vs. 89% Del(17p): 45% vs. 45% Del(11q): 62% vs. 66% TP53 mutation: 37% vs. 42%	mPFS: 38.4 vs. 38.4 m	NA
BRUIN CLL-321 [72]	Pirto vs. R + idela or R + benda	R/R CLL	Pts aged 18 years and higher	238	67 (42–90)	U-IGHV: 92.8% vs. 79.6% Del(17p): 46.2% vs. 44.5%	Follow-up of 11.6 m, HR of PFS: 0.55	NA

Nb: number; U-MRD: undetectable minimal residual disease; PB: peripheral blood; BM: bone marrow; y: years; m: months; Ibru: ibrutinib; Clb: chlorambucil; Obn: obinutuzumab; mPFS: median progression-free survival; NA: not available; NR: not reached; U-IGHV: unmutated immunoglobulin heavy chain; Del(11q): deletion 11q; Del(17p): deletion 17p; benda: bendamustine; R: rituximab; acala: acalabrutinib; Zanu: zanubrutinib; idela: idelalisib; Pirto: pirtobrutinib; HR: hazard ratio; R/R CLL: relapsed and/or refractory chronic lymphocytic leukemia; Pts: patients; FCR: fludarabine, cyclophosphamide, rituximab.

**Table 2:** Major phase III trials of combination of bruton tyrosine kinase inhibitors and BCL-2 inhibitors.

Trial	Treatment Arms	Line of Treatment	Population	Nb of Pts	Median Age (y) (Range)	Disease Characteristics	Primary Endpoint	U-MRD Rate (PB or BM)
CLL14 [11,74]	Ven + Obn vs. Clb + Obn	1	Frail patients	432	72 y (41–89)	U-IGHV: 61% vs. 59% Del(17p): 9% vs. 7% Del(11q): 18% vs. 20% TP53 mutation: 11% vs. 8%	mPFS: 76.2 vs. 36.4 m (*p* < 0.0001)	EOT: 76% vs. 35% At month 60: 27.4% vs. 12.9%
CLL13 [75]	Ven + Obn vs. Ven + Obn + Ibru vs. Ven + R vs. FCR or R + benda	1	Pts aged 18 years and older	926	62 y (31–83) vs. 60 y (30–84) vs. 62 y (27–84) vs. 61 y (29–84)	U-IGHV: 57% vs. 53% vs. 57% vs. 57% Del(11q): 19% vs. 14% vs. 19% vs. 18% Del(17p) and TP53 mutation excluded	mPFS: NR vs. NR vs. 63.4 m vs. 59.2 m U-MRD at 15 months: 86.5% vs. 92.2% vs. 57% vs. 52%	U-MRD at 15 months: 86.5% vs. 92.2% vs. 57% vs. 52%
UK Flair [13]	Ibru + Ven vs. FCR	1	Fit for FCR	523	62 y (56–67)	U-IGHV: 47% vs. 53% Del(17p): excluded Del(13q): 34% vs. 38%	36-month PFS rate: 97.2% vs. 76.8% (*p* < 0.001)	PB: 1-year U-MRD: 47.5% vs. 66%; 5-year U-MRD: 92.7% vs. 67.9%
GLOW [82,83]	Ibru + ven vs. Clb + Obn	1	65 y or older Younger than 65 y with comorbidities	211	71 y (47–93)	U-IGHV: 52% vs. 51% Del(17p): excluded Del(11q): 19% vs. 17% TP53 mutation: 7% vs. 2%	mPFS: NR vs. 21 m (*p* < 0.001) 42-month PFS rate: 74.6% vs. 24.8%	PB 3 months after EOT: 54.7% vs. 39%
AMPLIFY [86]	Acala + Ven vs. Acala + Ven + Obn vs. FCR or benda + R	1	Pts aged 18 years and higher	867	61 (26–86)	U-IGHV: 58.6% Del(17p): excluded	mPFS: NR vs. NR vs. 47.8 m 36-month PFS rate: 76.5% vs. 83.1% vs. 66.5%	

Nb: number; U-MRD: undetectable minimal residual disease; PB: peripheral blood; BM: bone marrow; y: years; m: months; Ibru: ibrutinib; Clb: chlorambucil; Obn: obinutuzumab; mPFS: median progression-free survival; NR: not reached; U-IGHV: unmutated immunoglobulin heavy chain; Del(11q): deletion 11q; Del(17p): deletion 17p; benda: bendamustine; R: rituximab; Acala: acalabrutinib; Pts: patients; ven: venetoclax; FCR: fludarabine, cyclophosphamide, rituximab; EOT: end of treatment.

**Table 3:** Major ongoing phase III trials in previously untreated or relapsed and/or refractory chronic lymphocytic leukemia.

Trial	Nb of Pts	Population	Arms	Primary Endpoint
ECOG-ACRIN EA9161 (NCT03701282)	720	Previously untreated CLL	Investigational: ibrutinib + venetoclax + obinutuzumab (IOV) Control: ibrutinib + obinutuzumab (IO)	PFS
MAJIC (NCT05057494)	607	Previously untreated CLL/SLL	Arm A: acalabrutinib + venetoclax Arm B: acalabrutinib + obinutuzumab	PFS
CLL16 (NCT05197192)	650	Previously untreated high-risk CLL defined as at least del(17p), TP53 mutation or complex karyotype	Arm A: acalabrutinib + venetoclax + obinutuzumab (GAVe) Arm B: venetoclax + obinutuzumab (GVe)	PFS
CLL17 (NCT04608318)	897	Previously untreated CLL	Arm A: Ibrutinib Arm B: venetoclax + obinutuzumab Arm C: venetoclax + ibrutinib	PFS
BRUIN CLL-313 (NCT05023980)	250	Previously untreated CLL/SLL	Arm A: pirtobrutinib Arm B: bendamustine + rituximab	PFS
BRUIN CLL-314 (NCT05254743)	650	Previously untreated CLL/SLL	Experimental: pirtobrutinib Control: Ibrutinib	ORR
GLORA-2 (NCT06319456)	344	Previously untreated CLL/SLL	Experimental: lisaftoclax + acalabrutinib Control: FCR	PFS
BELLWAVE-011 (NCT06136559)	1200	Previously untreated CLL/SLL	Experimental: nemtabrutinib Control: ibrutinib or acalabrutinib	ORR PFS
BGB-11417-301 (NCT06073821)	640	Previously untreated CLL	Experimental: zanubrutinib + sonrotoclax Control: venetoclax + obinutuzumab	PFS
BELLWAVE-010 (NCT05947851)	720	R/R CLL/SLL following at least one prior therapy	Experimental: nemtabrutinib (MK-1026) + venetoclax Control: venetoclax + rituximab	PFS
BRUIN-CLL-322 (NCT05254743)	600	R/R CLL/SLL	Experimental: venetoclax + rituximab + pirtobrutinib Control: venetoclax + rituximab	PFS

R/R: relapsed and/or refractory; CLL: chronic lymphocytic leukemia; SLL: small lymphocytic lymphoma; PFS: progression-free survival; ORR: objective response rate.
